# Development of Evaluation System for Iranian Health Research Networks: Challenges and Lessons Learned

**Published:** 2020-01

**Authors:** Shirin DJALALINIA, Mohammad Bagher TALEI, Abbas NAJJARI, Mohammad Reza BAGHERI, Shahin AKHONDZADEH, Reza MALEKZADEH, Asghar EBADIFAR

**Affiliations:** 1 Deputy of Research and Technology, Ministry of Health and Medical Education, Tehran, Iran; 2 Non-Communicable Diseases Research Center, Endocrinology and Metabolism Population Sciences Institute, Tehran University of Medical Sciences, Tehran, Iran; 3 Psychiatric Research Center, Roozbeh Hospital, Tehran University of Medical Sciences, Tehran, Iran; 4 Digestive Diseases Research Center, Shariati Hospital, Tehran University of Medical Sciences, Tehran, Iran; 5 Dentofacial Deformities Research Center, Research Institute of Dental Sciences, Shahid Beheshti University of Medical Sciences, Tehran, Iran

**Keywords:** Research network, Health, Evaluation, Iran

## Abstract

**Background:**

Health research networks (HRNs) are critical components of large-scale systems of production and validation of scientific evidence. As evaluation of research systems is a reliable process to measure efficiency and effectiveness of their activities, we aimed to report the processes of development of evaluation indicators’ for Iranian health research networks and the results of conducted assessment.

**Methods:**

In 2017, for the first time, aim to develop the evaluation framework for national HRNs, following the qualitative approach to assess the quality of research we designed the peer review method as one of the most important tools. This qualitative method was conducted according to experts’ views in specific fields. Key policy makers and stakeholders collaboratively developed a number of criteria for evaluation of research performance of Iranian HRNs. Following the review of conducted studies, benefitting from published guide line, these indicators were defined under 4 main axes of governance and leadership; infrastructures; research products and research impact.

**Results:**

Based on requirements of developed protocol for evaluation of HRNs in Iran, 18 HRNs completed the processes of evaluation. Results show a progressive need for more attention to precise planning of HRNs for achieving to goals. Another point to consider is the attention to documenting processes. The observational system for researches for detection of latest research priority was the most important issues that need to be more addressed by all of networks.

**Conclusion:**

Research evaluation of Iranian HRNs more over creating of constructive positive competition provide an overview of the shortcomings and research challenges could be used for better planning and promotion of the health research system.

## Introduction

Evaluation and monitoring the reliable evidence of research systems contributed to better policy decisions and promotion of health management (1, 2). Health research networks are critical components of large-scale systems of production and validation of scientific evidence (3, 4). The health network mostly commit to joint and structure a comprehensive setting of individuals, or institutions (such as universities, hospitals, institutes and other-related centers) under the predefined common mission. In health research networks, the main visions and strategies focus on require research plans. On the other hand, collaborative research networks are often touted as a solution for enhancing the translation of knowledge (5, 6). In Iran, the health research networks (HRNs) as the highest scientific level of confirmation of scientific evidence, play a considerable role in achieving the goals of the national targeted plans for health researches (6, 7). These virtual frameworks develop based on cooperation of a series of governmental and non-governmental research centers and research institutions in line with relevant goals to improve the quantity and quality of health research products (7–9). These research centers arranged contributed as 20 interactive research networks that are working under the supervision of the Deputy of Research and Technology of Ministry of Health and Medical Education (MOHME) (10).

Related studies on evaluation of research networks in other countries show that monitoring of research performance provide very useful information for promotion of health system research (11–13).

Regarding the evaluation of research networks, studies are scattered and mainly limited to clinical service provider networks (11, 14). They mostly discussed on different approaches of evaluation and emphasized of participatory plans that involve all related stakeholders (9, 15, 16). Related evidence emphasize on qualitative evaluation and peer review techniques as of the most important approach of research evaluation (9, 17).

In Iran, despite of importance and priority of problem, there is not any record of evaluation of research networks. Most of released results focused on evaluation of research performance and different approaches in evaluation of medical universities and health related research centers (18–20).

As evaluation of research systems is a reliable process to measure efficiency and effectiveness of their research performance, present paper reports the processes of develop the first indicators of evaluation of HRNs in Iran and results of evaluation. This included the identification of participants, development of indicators, barriers and enabling factors for their involvement in a participatory collaborative virtual research networks and extracted results of running the evaluation.

## Materials and Methods

In 2017, for the first time, we planned for pilot the evaluation of national HRNs. Following the qualitative approach we designed the peer review method as one of the most important tools. This qualitative method conducted according to experts’ views in specific fields.

A committee composed of experts of research fields along with the researchers of core team in MOHME, reviewed the provided research documents of each of Iranian HRNs. Moreover field visits conducted for assessment of equipment and facilities targeted by indicators of check lists. After aggregation of results, a descriptive report including analysis of strengths and weaknesses of research networks and suggestions for better promotion drafted by peers.

The Iranian HRNs were considered as evaluation units. The inclusion criteria were having approval from the Medical Council for the Development of Medical Sciences Universities (Table 1).

**Table 1:** The list of active Iranian Health Networks (by 2017)

***No***	***The Network title***	***The year of establishment***
1	The Molecular Medicine Network	2000
2	The Pharmaceutical sciences Network	2005
3	The Mental Health Network	2006
4	The Neuro Sciences Network	2006
5	The Ophthalmology Network	2006
6	The Censer Network	2006
7	The Leishmaniasis Network	2010
8	The Hepatitis Network	2010
9	The Dental and Oral Disease Network	2010
10	The Diabetes Network	2010
11	The Osteoporosis Network	2010
12	The Nanotechnology Network	2011
13	The Spinal Injury Network	2011
14	The Cardiovascular Disease Network	2011
15	The Respiratory Disease Network	2011
16	The Environmental Health Network	2011
17	The Lasers in medicine Network	2011
18	The Medical Biotechnology Network	2011
19	The Cohort Research Network	2016
20	The nursing Research Network	2016

### Development of the scientific structure of the study

Under the supervision of MOHME, the scientific committee was established with participation of core research team, leading experts of evaluation systems and scientific referees of clinical and biomedical fields.

### Designing the indicators

Providing the results of primary review, using expert panels, the peer-based evaluation indicators designed. During 4 sessions, a list of objective-oriented evaluation indicators set for evaluation of research flow of networks.

Based on the reported successful experiences, the interested guideline of peer-based research evaluation model selected and developed indicators defined under 4 main axes of this guideline including; governance and leadership (priority setting of researches, strategic plan, the stakeholders’ analysis, executive plans, network secretariat), infrastructures (website, fundraising, resources management), research products (clinical guide lines and Instructions, observational system for researches) and research impact (national production of health and technologies) (15, 16).

### Weighting the indicators

After assessing different approaches and methods discussed for appropriate weighting and aggregation of scores, the weighting of scores of indicators set based on the main policies, and sustainability perspectives (21, 22). After determination of the weight of each of evaluating axis, the weighting of their indicators was determined.

### Primary assessment of evaluation form

The results and executive challenges of primary assessment of indicators followed through pilot study in 4 networks. After consideration of feedbacks and required revisions, final version approved for main evaluation.

### Finalizing the protocol of evaluation of Iranian HRNs

Final version of indicators completed in the form of integrated protocol of evaluation of Iranian HRNs included the main evaluating axis, targeted criteria along with corresponding definitions, and the complementary documents (Table 2).

**Table 2:** Axes and Criteria for IHRNs Evaluation

***No***	***Axis***	***Criteria***	***Definition***	***Expected documents***
1	**Governance and leadership**	Priority setting of researches	The process and strategies of selection of health researches /interventions.	— The list of network research priorities — Documentation of processes and methods
		Strategic plan	The network process of defining its strategies, or directions, and making decisions on allocating its resources to pursue this strategies.	— Running strategic plan — Documentation of the process of development or updating of strategic plan — Approval commitment of the scientific council of the network — Documentation of processes and results of monitoring and evaluating of in process strategic plan
		The stakeholders analysis	This information is used to assess how the interests of those stake-holders should be addressed in a project plan, policy, program, or other actions of network.	— The list of potential and actual stakeholders — Documentation of attract participation determination of common interests — The framework of stakeholder analysis according to the type of internal and external partnership, the intensity of the impact and the importance of participating in network affairs — Inter-departmental and outsourced collaboration executive programs
		Executive plans	The time binding predefined documented programs for address the executive plans of network strategic plan	— Providing an operational plan in accordance with the strategic plan of the network — Documentation of the implementation of the activities contained in the program — Documentation of evaluation and feedback of implemented programs
		Network secretariat	Documentation processes of the network secretariat activities	— Documentations of meetings and events — Reports on the progress of network research projects processing — Introduction of research network and related advocacy documents
2	**Infrastructures**	Updated website	A set of related web pages located under a single domain of network	— Active and up-to-date website — Content of the website (network introduction, programs, members, news, engagement of stakeholders, annual calendar, statistics of visitors ...)
		Fundraising	Fundraising from other organizations except from the Ministry of Health	— Proposals or approved by the network research council — Contract or memorandum of cooperation — Documentation of the transfer of funds or goods or services
		Resources management	The ratio of the spent budget to the allocated budget	— Financial report and documentation
3	**Research products**	Clinical guide lines and Instructions	Clinical guide line / Instructions for implementation / nationalization of products or services	— Documentation of related processes including proposals, decisions of the research council of the Network — Approval and communication of credit and scientific application by the highest competent authority of the relevant deputy of the Ministry of Health — Articles and other outcomes of the research
		Observational system for research	The existence of a system for observing scientific developments in the field of activity or research priorities of the network	— Documentation of system performance — Reports extracted by the system — Documentation of the use of reports and the dissemination of results to stakeholders
4	**Research impact**	Supporting national production of health and technologies	Program for supporting national health and technologies	— Documentation of relevant processes including proposal, approval of the research council of the network — Approval and communication of credit and scientific application by the highest competent authority of the relevant deputy of the Ministry of Health — Agreements and commitments between researchers and users

Before the official start of the evaluation, aim to reach common understanding of processes and require cooperation, we conducted a participatory training workshop for focal points of Iranian HRNs, through which the justification of recent evaluation and the scientific process discussed.

### Implementation of peer reviews

**Fig. 1:**
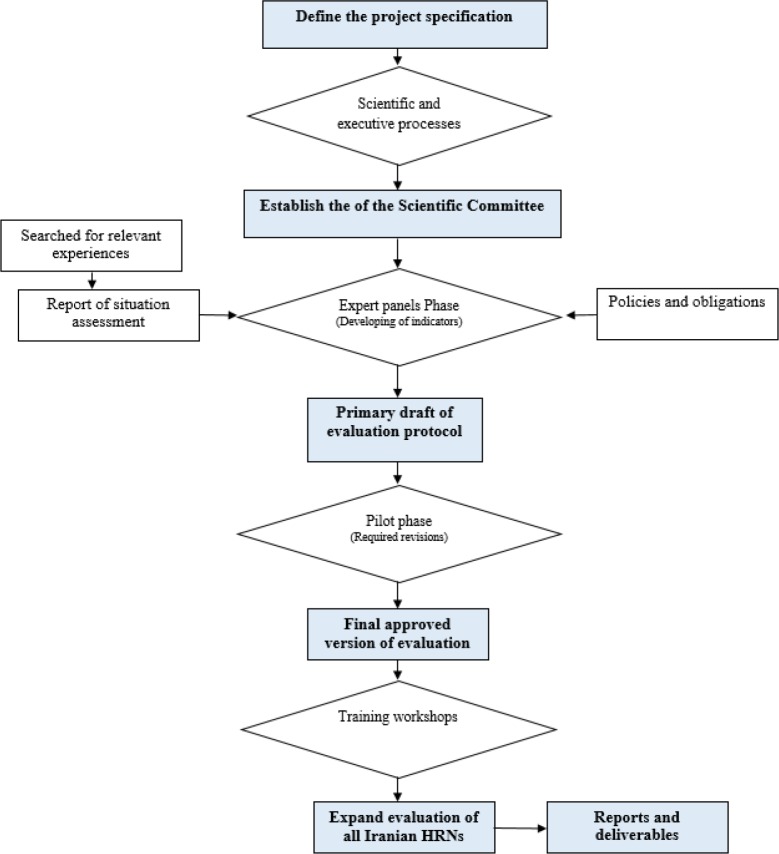
Flowchart of processes of the evaluation of Iranian HRNs

Received completed forms and related documents evaluated through the peer review sessions by core research team and scientific referees. The field visits conducted for assessment of equipment and facilities of each of HRNs (2 networks did not attend the evaluation). Figure 1 shows the processes of development of the evaluation criteria and evaluation of Iranian HRNs.

## Results

One of the main deliverable of present investigation is developed protocol and indicators of evaluation of research performance in Iranian HRNs. Out of the twenty networks, approved at the time of the study, 18 HRNs completed the processes of evaluation. Based on four main axis the results of analysis presented as follows:

**a. Governance and leadership:**
  ***a.1. Priority setting:*** Considering the standard defined process of priority settings, many did not go through the prioritization of research topics. In few cases, the availability of resources and research interests of main partners specifies the direction of the research
  ***a.2. Strategic planning:*** Documentation of development of plan mostly was less than favorable standard. Except for two networks, in majority of them the objectives and plans were not followed according to predefined the strategic plan. According to evaluation of documents, even, for two networks, the targeted vision and planed mission were not specific.
  ***a.3.The stakeholders’ analysis:*** The process of assessing a system and potential changes to it as they relate to relevant and interested parties, was only done by one of the networks. In other cases, it was merely to provide a simple list of current contributors to the activities and collaborative projects.
  ***A.4.Executive plans:*** The mission statements were neglected in drafting the executive plan of three networks. As another point; in many networks the setting of ongoing plans were not did not completely match with the extracted objectives of their strategic plan. Practically in most of cases, the annual evaluation of programs was the most evident gap.
  ***A.5.Activities of network secretariat:*** This topic evaluated the processes of interactive performance of the network secretariat with other institutions and stakeholders. This approach was focused mainly on coordination of inter-action activities and follow-up of legal requirements. From this point of view most of networks have set up a good executive structure for the secretariat.
**a. Infrastructures**
  ***B.1.Updated website:*** Fortunately, the principles and standards of both; technical design and content development were predicted by a considerable 13 number of networks. Meanwhile, as an essential and important challenge, the website were missing gap in three networks.
  ***b.2.Fundraising:*** Only in few networks we detected the acceptable results in fundraising. According to this, few successful cases of gathering voluntary contributions of money or other resources had occurred based on the specific key roles of handful number of key leaders.
  ***b.3.Resourcesmanagement:*** Although most of networks are experiencing problems in attracting the new and efficient resources, the mechanisms and processes of attracting and spending the current allocations of the Ministry of Health work efficiently.
**b. Research products**
  ***C.1.Clinical guide lines and Instructions:*** Overall 11 national clinical guide lines were documentedin8 involved networks.
  ***C.2.Research observation systems:*** Eight networks provided related document to their infrastructures and planning for research observation system. Some of them were designed and managed based on traditional approaches of periodic situation analysis that mainly conducted through systematic reviews. One of the networks planned for comprehensive setting of electronic infrastructure that provides practical specific reports according to different target groups.
**c. Research impact**

The results show that we are faced with a reliable gap in all of networks. This challenge overwhelm all aspects of the problem from first steps of policy making to practical plans of implementation.

## Discussion

The present study, is the first assessment of research performance of Iranian health research networks conducted according to the qualitative method that set based on experts’ views in specific fields.

Through this experience of peer review, following completed the processes of evaluation, based on the extracted results required feedback reflected to the networks. The assessed indicator developed under 4 main axes of; governance and leadership, infrastructures, research products, and research impact that each of them was evaluated based on detailed scoring of sub-categories. Our findings emphasize on the progressive need for more attention to precise prioritized programs which can lead to mission-oriented health research networks.

Although there is a large body of evidence on practical concepts of the research networks in health domains and research fields, the conceptualizations and implementations of monitoring and evaluation of their activities remain controversial challenge (12, 13). Distributed results mainly focused on clinical and health providing networks (11–13, 23).

In most of other communities research networks compose of funders, policy makers, and research organization aimed at improving specific outcomes (13, 24). From this point of view, collaborative planning and activities, effective team working, interactive communication, sharing of facilities and capacity building should be considered as essential and integral components of productive research networks (13, 24).

In our country studies in this field, have mainly focused on the evaluation of research trends and the evaluation of research activities of medical universities and medical research centers (9, 18–20).

In Iran, peer review evaluation model works based on 4 main axes of governance and leadership; structure; knowledge production and research impact (9). Considering the field of evaluation, each axis is assessed through several indicators. This approach is also consistent with “Excellence in Research for Australia” model (ERA) (15). Through this model research activities (number of students, research budget, number of academic members, etc.); quality of research (number of publications, number of citations, etc.); and applied research quality (revenue from research, patents, etc.) consider as the main axis of evaluation(15, 16).

The related studies on evaluation of HRNs are limited. This growing field of research, in new expanding scope mainly focus on research missions, intends to assess and provide best practices for effective planning and evaluation tool. In this regards, peer review methods not only facilitate the evaluation process of network settings, but engage the leadership and members in a progressive productive process (9, 23, 25).

Considering the priorities, after comprehensive arrangement of all required components for strategic planning and scientific management of policies and programs, another specific attention should be focused on precise observing and documenting the processes and deliverables. This would undoubtedly be tailored in the best way through empowering the network administrators and executive partners (11, 14).

Following the passing of near two decade of development of research networks in Iran, in view of the mission and objectives of the research networks, the need for quantitative development and the improvement of the quality of their activities, become one of the top interests of national research management programs (10, 14).

Related scientific resources reveal that; given the vision and missions of the network, it is necessary to consider the complementary quantitative and qualitative assessments of research outcomes, as well as the impact of research on community health promotion (9, 12, 26, 27).

Given the rapid growth of knowledge, especially in sensitive and specific research areas, the prediction and utilization of up-to-date information observing systems is one of the most important issues that need to be addressed with greater commitment and focus. Such key data collection system should be responsible for keeping up-to-date observing and detecting of the latest information and findings of interested fields of research networks activity (27, 28).

Parallel with all of scientific stakeholders and target consumers, as the main level of policy-making and professional reference, HRNs should be involved in all stages of clinical instructors and medical guidelines development.

In our assessment programs, ongoing plans for supporting and even managing the national health and technologies were the other important topic followed for evaluating the quality of researches in networks (9, 29).

Considering the implication of our finding in the field of policy and management, we need to a prompt action for involving the research networks in national planning and supporting the large scale health research and critical technologies (11, 29).

Many fundamental barriers of optimal health research networks performance including; the fragmented non-relevant research policies, insufficient resources management, and some cultural problems, have been studied through the related investigation.

As the main strength, present paper reports the first experience of evaluation of research activities of Iranian health research networks. We developed a practical criteria cover the main axis of health system researches. For more exact results we planned for interactive processes of review and completing the documents.

We also faced with some limitations. As the first round of evaluation we mainly focused on leadership and simple indicators of infrastructures

As it was the first experience of peer-based evaluation of HRNs in Iran, we inevitably restricted the indicators to more tangible items. Definitely, in the continuation and more development of this evaluation, the indicators should be designed to meet the highest expected standards.

We need to more advocacy and serious set up for assignment of national missions to national HRNs. Only then we will be able to be expected for detecting the improvement of quantity and quality of research outcomes and research impacts. Accordingly, these indicators were not evaluated at this stage.

## Conclusion

Results show a progressive need for more attention to assessment the research performance of IHRNs. The research observing systems for detection of latest research priority was the most important issues that need to be more addressed by all of IHRNs.

Research evaluation of Iranian HRNs more over creating of constructive positive competition provide an overview of the shortcomings and research challenges could be used for better planning and promotion of the health research system. Further research is needed on complementary methods of evaluation and practical recommendation on national HRNs promotion.

## Ethical considerations

Ethical issues (Including plagiarism, Informed Consent, misconduct, data fabrication and/or falsification, double publication and/or submission, redundancy, etc.) have been completely observed by the authors.
